# EPIDEMIOLOGICAL PROFILE OF PATIENTS WITH OPEN FRACTURES (2019 TO 2020)

**DOI:** 10.1590/1413-785220243204e278000

**Published:** 2024-10-07

**Authors:** Rafhael da Costa Rodrigues, Marco Freire Vieira, Mário Augusto Ferreira Cruz, Lucas Vinícius da Fonseca Barreto, Rafael Chaves Souza

**Affiliations:** 1. Hospital Universitario de Lagarto, Ortopedia e traumatologia, Lagarto, SE, Brazil; 2. Empresa Brasileira de Serviços Hospitalares EBSERH-HUL, Ortopedia e traumatologia, Lagarto, SE, Brazil

**Keywords:** Fractures, Trauma, Open Fracture, Fratura, Trauma, Fratura Exposta

## Abstract

**Objective:**

To evaluate the epidemiological profile of open fractures treated at the University Hospital of Lagarto in the years 2019 and 2020.

**Methods:**

This is an observational, retrospective study, using data from electronic medical records.

**Results:**

In total, 312 patients met the inclusion criteria for this research and were included. The mean age of affected patients was 36.8 years. The main segment affected were the fingers, mostly affecting males (89%) and predominantly the left side (57.62%).

**Conclusions:**

The male sex was the most affected by open fractures, and the most prevalent trauma mechanism was motorcycle accidents. Moreover, we found that the fundamental criteria for care in open fracture cases were not always considered by the professionals, resulting in a lack of uniformity in the adopted procedures and discrepancies with the guidelines recommended in the specific literature. **Level of Evidence III, Comparative retrospective study.**

## RESUMO

**Objetivo:**

Avaliar o perfil epidemiológico das fraturas expostas atendidas no Hospital Universitário de Lagarto nos anos de 2019 e 2020.

**Métodos:**

Trata-se de um estudo observacional, retrospectivo, por análise de dados de prontuário médico eletrônico.

**Resultados:**

Foram incluídos 312 (trezentos e doze) pacientes que atenderam aos critérios de inclusão para esta pesquisa. A média de idade dos pacientes acometidos foi de 36,8 anos. O principal segmento acometido foram os dedos das mãos, conforme Tabela 1, em maior número no sexo masculino (89%) e predominando no lado esquerdo (57,62%).

**Conclusões:**

O gênero mais acometido por fraturas expostas foi o masculino, e o mecanismo de trauma mais prevalente foi o acidente motociclístico. Todavia, nem sempre os critérios foram levados em conta pelos profissionais, não apresentando homogeneidade nas condutas adotadas, bem como desencontro com as condutas orientadas na literatura específica. ***Nível de Evidência III, Estudo Retrospectivo Comparativo* .**

## INTRODUCTION

 Fracture is the result of failure of bone physical integrity and occurs when the force applied to the bone exceeds its resistance. This imbalance can occur due to the force being too great, or because the bone is weakened. An object under the action of a force undergoes deformation which, within certain limits, is reversible. However, if the force increases, the deformation reaches a critical limit at which the material will break, constituting a fracture, and the same principle applies for the bone. ^1^

 A fracture is considered open when the soft tissue envelope ruptures over or near the fracture site in a way that the underlying bone or fracture hematoma communicates with the external environment. ^2^ Moreover, when a fracture occurs in contaminated cavities, such as the digestive and genitourinary systems, it should be considered exposed. ^3^ Thus, as noted by Court-Brown et al., ^4^ treating open fractures requires a multidisciplinary approach rather than relying on a single specialty to achieve better patient outcomes. 

 Open fractures (OF) are usually caused by high energy trauma, with car accidents being the most common. ^5^ It has a preferential distribution in the age group ranging from the second to the fourth decade of life, with a higher prevalence in men. ^6^ The bones located in the lower limb are the ones that suffer the most from this type of injury, with the tibia being the most affected bone. ^3^

 In some cases, diagnosing open fractures may be challenging, as observed by Filho et al., ^7^ who state that the diagnosis of OF can be difficult since the communication point of the skin lesion may be distant from the fracture focus, or even be minimal or imperceptible. Thus, whenever a tissue injury is evidenced in the fracture segment, the possibility of OF should be considered. This type of injury becomes more serious among fractures due to the various complications they can entail, with the increased risk of infection, loss of limb function, and neurovascular injuries. ^2^

 However, several classifications of OF correlate the bone fracture/soft tissue binomial to evaluate prognosis and determine the most appropriate treatment. ^8^ Currently, the most widespread is the Gustilo and Anderson classification, which considers the kinetic energy of the trauma, time of exposure, affected segment, severity of the soft tissue injury, characteristics of the fracture, neurovascular status, and degree of contamination. ^7^

 Time is paramount in relation to the clinical outcome of the fracture. Torneta III et al. (2019), ^2^ emphasizes that the ideal time from the moment of fracture to the surgical approach should not exceed six hours after the injury, considering that after this period there may be an increased risk of infection at the site. Nevertheless, antibiotic therapy should be started as early as possible, as it is the main factor in preventing infection. Moreover, Hebert et al. stated that the main goal of the treatment of open fractures is to prevent infection, obtain adequate bone union, and heal soft tissues, leading to functional recovery of the affected limb as early as possible. ^8^

Based on these aspects and considering that epidemiological studies are essential to develop an understanding of the pathology and aid in therapy and preventive measures, this study aimed to evaluate the epidemiological profile of open fractures treated at the university hospital of Lagarto in the years of 2019 and 2020.

## MATERIALS AND METHODS

Retrospective observational studies were conducted. Data were obtained from patients treated by the orthopedics team at the Emergency Unit of the University Hospital of Lagarto from January 1, 2019, to December 31, 2020, using the institution’s database—collection conducted via electronic medical records. This study was approved by the Research Ethics Committee on Human Beings of the Federal University of Sergipe (UFSLAG/HUL), under CAAE: 61267522.1.0000.0217 and opinion number 5.823.198. Participants signed an informed consent form.

Information regarding sex, age at the time of the initial evaluation, fracture aspects (mechanism of injury, location, presence of contact with the external environment), classification according to the Gustilo and Anderson classification, and time elapsed since the first orthopedic treatment to the initial approach. Moreover, data related to the radiographic evaluation of the imaging exams present in the electronic medical records were collected from the hospital database and inserted into the study.

The study included participants over 18 years of age, undergoing orthopedic treatment for at least two months, with at least one regular weekly frequency at the hospital, and a minimum 20 minutes per workout.

The data were stored in a spreadsheet and studied using the Excel software (Microsoft). A descriptive analysis was performed using measures of central tendency (mean, median), variability (standard deviation), and position (maximum and minimum).

## RESULTS

A total of 320 medical records of patients treated at the University Hospital of Lagarto of the Federal University of Sergipe (HUL-UFS) from January 1, 2019, to December 31, 2020, with possible open fractures were analyzed. Of these, 312 met the inclusion criteria for this research. Of the eight medical records excluded, three did not present specific data defining whether the injury was a true open fracture, in two cases referred by general surgery, orthopedics ruled out open fractures, and in three cases the patients were treated in another emergency service.

The average number of open fractures treated during this period was 0.42 patients per day, i.e., about one patient every 2.4 days. The day with highest attendance was November 20, 2020, a Sunday with four cases of open fractures.

Considering the annual frequency, we found 148 cases of open fractures in 2019, with the most affected site being the fingers with 53 cases, followed by the toes with 33 cases and the tibia with 15 cases. In 2020, we found an increase in the number of OF cases in the order of 10.81% compared to 2019, totaling 164 OF cases. The most affected sites did not change, but we found a higher number of open fractures of the tibia than of the toes: fingers (65 cases), tibia (29 cases), and toes (28 cases).

Considering the studied population, we found a higher prevalence of open fractures in men 265 (85%) than in women 47 (15%). Regarding the age group, individuals in the third decade of life, from 21 to 30 years old, were the most affected, with a total of 68 patients.

The mean age of the affected patients was 36.8 years and standard deviation (SD) was 17.55 years, in a population of patients ranging from 3 to 90 years. The most affected age was 53 years, corresponding to 13 cases (4.1%).

Regarding the trauma mechanisms related to the OF, we found 12 causes, in the following order of prevalence: motorcycle accident (46.8%), cutting machine (marble saw, chaff cutting machine, chainsaw, etc.) (28.2%), white weapon injury (WWI; knife, machete, hatchet, etc.) (9%), fall from the same height (6.1%), run-over (2.9%), gunshot injury (GI; 1.9%), animal-drawn transport (cart, horse, etc.) (1.9%), bicycle accident (1.3%), fall from great height (scaffolding, ladder, tree, etc.) (1%), automobile accident (0.6%), and animal bite (0.3%).

The most affected side during open fractures was the left side. During the analyzed period, we found no bilateral open fracture.

 To study the location of the open fractures, it was necessary to divide them into segments, as shown in Table 1 . 

**Table 1.**
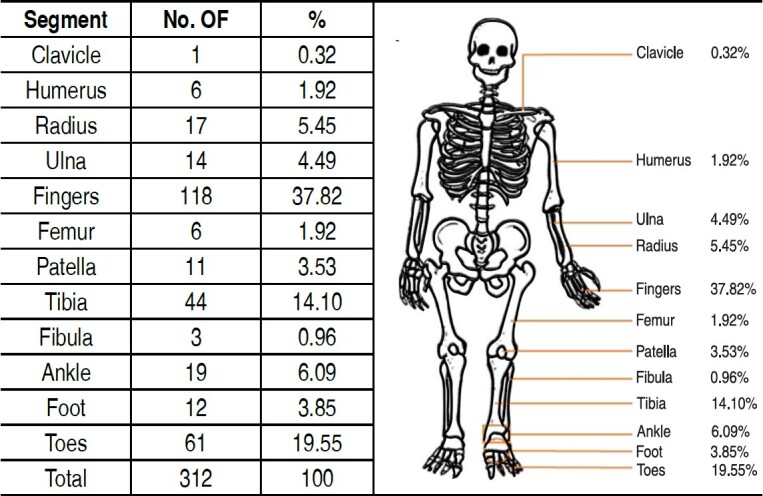
Frequency of open fractures by body segment over the analyzed period.

**Source** : prepared by the authors (2024)

**Graph 1.**
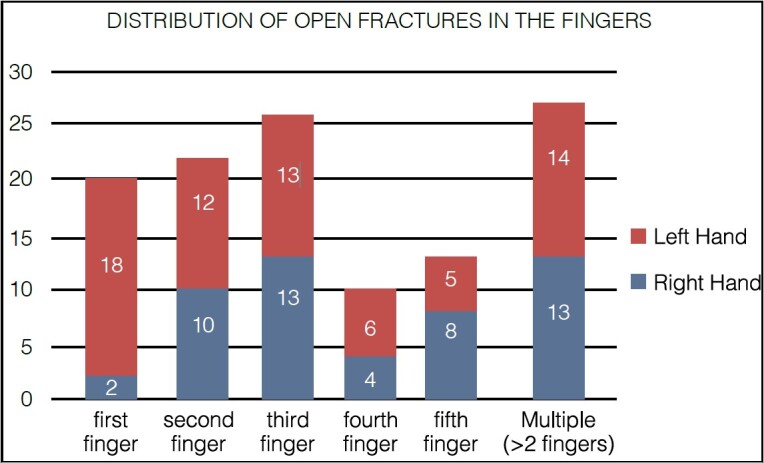
Distribution of open fractures in the fingers.

 The most affected segment was the fingers, which showed greater numbers in men (89%) and predominantly on the left side (57.62%). Graph 1 shows the frequency of finger involvement. 

**Source** : prepared by the authors (2024)

The mechanisms of hand finger injuries found in order of occurrence were cutting machines (marble saw, chaff cutting machine, chainsaw, etc.) 79 cases (66.95%), white weapon injury (WWI) 26 cases (22.03%), motorcycle accident 9 cases (7.63%), gunshot injury (GI) 2 cases (1.69%), and automobile accident and animal bite each with one case (1.7%).

Regarding multiple injuries, in which there are more than two fingers affected by an open fracture, we found 27 cases. The main mechanism of trauma in this situation was accidents with cutting machines (marble saws, marble, chaff cutting machine, chainsaws, etc.), which occurred in 11 cases, representing 40.74% of the cases of multiple exposed injuries in fingers.

When considering only the long bones, the highest incidence of open fractures was in the tibia (44 cases), being more prevalent in males (37 cases) with a higher number of injuries on the right side (24 cases). The most common mechanism in this situation was motorcycle accidents, accounting for 34 cases (77.3%) of open fractures in the tibia, followed by run-overs in eight cases (18.2%). In the upper limbs, the most affected bone was the radius (17 cases), being the second most affected long bone, also with a predominance in males (12 cases) and most often injuring the left side (13 cases). In this case, the main mechanism of injury was also motorcycle accidents (9 cases), followed by falls from same height (7 cases).

**Graph 2.**
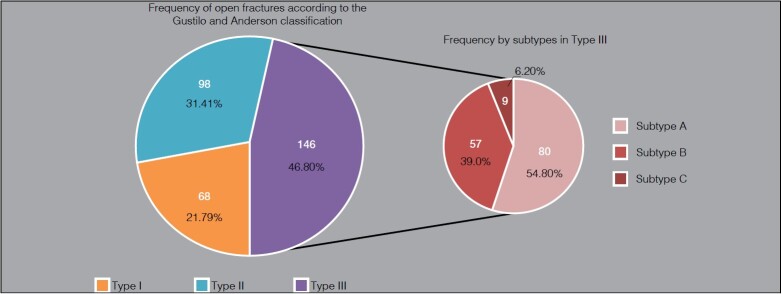
Frequency of open fractures according to the Gustilo and Anderson classification.

 Using the Gustilo and Anderson classification as a basis, the most frequent open fracture was grade III ( Graph 2 ). Graph 2 shows the distribution of open fracture types based on the Gustilo and Anderson classification. 

Table 2 shows the frequency of open fractures by anatomical site according to the Gustilo and Anderson classification. 

**Source** : prepared by the authors (2024)

**Table 2.** Frequency of open fractures by site according to the Gustilo and Anderson classification

Segment	Gustilo and Anderson classification					
	Type I	Type II	Type III A	Type III B	Type III C	**Total**
Clavicle	1	0	0	0	0	**1**
Fingers	25	23	38	27	5	**118**
Toes	11	35	9	5	1	**61**
Femur	0	0	4	2	0	**6**
Fibula	2	1	0	0	0	**3**
Patella	0	9	0	2	0	**11**
Foot	1	4	0	7	0	**12**
Radius	10	4	2	1	0	**17**
Tibia	1	13	23	7	0	**44**
Ankle	9	4	3	3	0	**19**
Ulna	8	4	0	2	0	**14**
Humerus	0	1	1	1	3	**6**
**Total**	**68**	**98**	**80**	**57**	**9**	**312**

**Source** : prepared by the authors (2024)

## DISCUSSION

In this study, we analyzed the epidemiology of 312 open fractures retrospectively in the period from January 1, 2019, to December 31, 2020, to identify the numerous aspects of this type of injury and thus reinforce our conviction that the study of these injuries is of vital importance for our institution, as well as the community.

The choice to analyze data from a two-year period was due to the scarcity of other epidemiological studies in the service, hindering possible comparisons and prospective statistics. We also aimed to assess possible changes in epidemiological aspects, given that the year 2020 was atypical due to the COVID-19 pandemic.

 When comparing both years, 2019 showed 148 cases (47.44%) of open fractures, while 2020 showed 164 cases (52.56%). We observed a 10.8% increase in the number of OF cases in 2020 compared to the previous year. This increase was mainly due to accidents involving motorcycles, drawing attention since it is associated with the increase in the number of delivery drivers in the pandemic peak; however, no reliable static data can prove this theory. In a study conducted by Cunha et al., an average of 4.96 cases/day was found. Our study showed an average attendance of 0.42 patients/day with open fractures, that is, about one patient every 2.4 days. ^9^

 Court-Brown et al. show that the frequency of open fractures in long bones is 11.5 per 100,000 inhabitants. ^4^ Based on the state health plan of the government of Sergipe from 2016 to 2019, the University Hospital of Lagarto serves an estimated population of 257,633 inhabitants, so the frequency of open fractures in long bones in this service during the study period was 19.41 per 100,000 inhabitants in 2019 and 22.46 per 100,000 inhabitants in 2020. This distortion in incidence and frequency when compared to the literature may occur from region to region, depending on the characteristics of the sample, their economic activity and socio-educational conditions, means of transportation and traffic laws, and occupational safety standards and their inspection, that is, several regional variants. ^3^ Based on these variables, Court-Brown et al. state that developing countries, such as Brazil, show higher rates of OF in long bones per 100,000 inhabitants due to a higher number of traffic accidents, especially motorcycle accidents, as well as accidents in the workplace, ^4^ which corroborate our study. 

 As demonstrated in the study by Arruda et al., in which the left side was predominant in 59.06% patients, the greatest laterality in our study was also on the left side with 170 cases (54.48%). ^6^ This datum corroborates the relationship between the lack of agility in protecting the non-dominant limb, as most participants were right-handed. 

 Most OF occurred in male patients with a ratio of 5.5:1, in which 265 out of 312 cases occurred in this population, corresponding to 85% of the cases and corroborating other studies. Cunha et al. showed a prevalence of 84.2% of the cases being in males, ^9^ as well as Arruda et al., who presented a percentage of 86.84% of males affected in their study and a sex ratio of 6.6:1. ^6^ This result can be partially attributed to the greater exposure of men to various risks: the use of piercing and cutting utensils, psychological immaturity, a greater tendency to inexperience and disobedience to traffic rules, greater involvement with violence and fights, and greater labor exposure. 

 Patients in the third decade of life were the most affected by open fractures, accounting for 21.8% of cases (68 patients). A similar result was found by Arruda et al. and Cunha et al., ^6^^,^^9^ who found a mean age of 30 years (SD 16 years), with a mode of 21 years in the study by Arruda et al. and 25 years in the study by Cunha et al. ^6^^,^^9^ In our study, the mean age of the patients was slightly higher; however, it remained close to that demonstrated in the literature, being 36.8 years of age (SD 17.55 years). Our mode, on the other hand, differed quite differently from those presented and remained in the age of 53 years, corresponding to 4.1%, with 13 cases. This trend may have occurred due to greater recklessness in the use of machinery and sharp instruments by these patients, as they had been handling them for some years in their work activities, associated with less motor agility to deal with them compared to younger patients, affecting the fingers of the hands in greater numbers (8 cases). 

 The segment with the highest frequency of occurrence of open fractures in this study was the fingers (37.8%), a fact also found in the study by Cunha et al., ^9^ with OF of the bones of the hands representing 27.6% of the total open fractures. 

 According to Court-Brown et al., tibial shaft open fractures are the most common among long bones. ^4^ This has been also found in the study by Hanciau ^3^ , in which the tibia represented 21.6% of OF cases, and by Arruda et al., in which they found 37.86% frequency in this bone segment. ^6^ Therefore, if we consider only long bones, the highest percentage of involvement can be found for the tibia, representing 48.35% of the cases, which demonstrates the balance of our service with the data found in the literature. 

 Arruda et al. mention in their study that the use of motorcycles with greater exposure of the lower limbs contributed to the greater number of open fractures in these segments. ^6^ Our research found 146 cases of open fractures caused by motorcycle accidents, accounting for 46.8%, being the most common mechanism of trauma. For Hanciau, knowledge of the trauma mechanisms that lead to OF serves as a guide to alert us to carefully look for injuries, including obscure ones. ^3^

 We found that the most common trauma mechanism for open fractures occurred on public roads, accounting for 53.52%, which is similar to the findings by Arruda et al., in which 57.30% of cases were found. These included being run over, car, motorcycle, and cycling accidents, as well as accidents involving animal traction, horses, and carts. ^6^

Injuries caused by cutting machines (marble saws, marble, chaff cutting machine, chainsaws, etc.) caused open fractures and accounted for the third highest incidence, with 31 cases (9.9%), predominantly in fingers (30 cases). We believe that this fact may have occurred due to the region covered by the hospital showing an economy predominantly linked to rural areas, which use a lot of machinery of this type and where their operators have low schooling and little training to use such equipment, increasing the chances of accidents.

In our study, open fractures in the fingers were the most common (118 cases), with the third finger being the most frequently affected (26 cases, 22.03%), followed by the second finger (22 cases, 18.64%), the first finger (thumb) (20 cases, 16.95%), the fifth toe (13 cases, 11.02%), and finally, the fourth finger (10 cases, 8.47%). On the other hand, we found 27 cases (22.88%) of the lesions involving multiple fingers (≥ 22.88%), which demonstrates more severe injuries due to improper handling of cutting machines. However, we did not find a relationship that could explain these numbers, believing them to be only fatalities.

 Our research was based on the Gustilo and Anderson classification for open fractures, considering the information provided in medical records. ^10^^-^^12^ Thus, we found that the highest incidence was in Type III, with 146 cases (46.79%). This result corroborates most other studies, as according to Arruda et al., the highest incidence was also found in Type III with 45.36%, a result close to that found in the study by Cunha et al., which revealed an incidence of 54% in this type of fracture. ^6^^,^^9^

 In our study, Gustilo and Anderson Type III presented the following results for its subdivision: in Type IIIa, we found the highest incidence with 80 cases (25.64%), followed by Type IIIb with 57 cases (18.27%) and Type IIIc, which showed nine cases of greater severity (2.88%). These findings are supported, in all proportions, by the study by Cunha et al. which shows similar figures: Type IIIa 48.6%; Type IIIb 3.5%; and Type IIIc 1.9%. ^9^ However, it differs from the study by Arruda et al. since they showed Type IIIa with 30%, Type IIIb with a lower incidence, only 5%, and Type IIIc with an incidence of 11%, which is justified by the fact that they conducted the study in a hospital of greater complexity which is a reference for more critical situations. ^6^

 Type I fractures showed an incidence of 68 (21.79%) cases, with the most prevalent being finger trauma with 25 cases, followed by toe trauma with 11 cases and radius with 10 cases. In Type II, we found 98 (31.41%) cases, with the toes being the most affected site in 35 cases, followed by fingers with 23 cases and tibia with 13 cases. Thus, we found that open fractures in our service are of a more severe nature, according to the Gustilo and Anderson classification. ^10^^-^^12^ This fact may be due to the hospital’s regional status and its role as a reference center for smaller units (emergency centers, small sized hospitals, basic health units, etc.), which results in it receiving most of the more severe injuries. 

Based on what was presented, it was observed that the male sex was the most affected by open fractures, and the most prevalent trauma mechanism was motorcycle accidents. Therefore, we can conclude that a direct approach to this population group would be vital to raise awareness about the severity of the situation, aiming to reduce the incidence.

As an orthopedic emergency, open fractures must be classified and evaluated based on four fundamental criteria: type of fracture, soft tissue damage, neurovascular compromise, and contamination potential. In this study, we observed that, across various medical records and practices, the professionals did not always consider the criteria, resulting in a lack of uniformity in the adopted procedures and discrepancies with the guidelines recommended in the specific literature. This conclusion was possible based on the difficulty encountered in analyzing and quantifying the information presented in the cataloged medical records.
